# Large Dosage of Chishao in Formulae for Cholestatic Hepatitis: A Systematic Review and Meta-Analysis

**DOI:** 10.1155/2014/328152

**Published:** 2014-06-02

**Authors:** Xiao Ma, Ji Wang, Xuan He, Yanling Zhao, Jiabo Wang, Ping Zhang, Yun Zhu, Lin Zhong, Quanfu Zheng, Xiaohe Xiao

**Affiliations:** ^1^Pharmacy College, Chengdu University of Traditional Chinese Medicine, Chengdu 611137, China; ^2^China Military Institute of Chinese Medicine, 302 Hospital of People's Liberation Army, Beijing 100039, China; ^3^Department of Evidence-Based Medicine and Clinical Epidemiology, West China Hospital, Sichuan University, Chengdu 610041, China; ^4^State Key Laboratory of Biotherapy, West China Hospital, Sichuan University, Chengdu 610041, China; ^5^Department of Integrative Medical Center, 302 Hospital of People's Liberation Army, Beijing 100039, China

## Abstract

*Objective*. To evaluate the efficacy and safety of large dosage of Chishao in formulae for treatment of cholestatic hepatitis. *Methods*. The major databases (PubMed, Embase, Cochrane Library, Chinese Biomedical Database Wanfang, VIP medicine information system, and China National Knowledge Infrastructure) were searched until January 2014. Randomized controlled trials (RCTs) of large dosage of Chishao in formulae that reported on publications in treatment of cholestatic hepatitis with total efficacy rate, together with the biochemical indices including alanine aminotransferase (ALT), aspartate aminotransferase (AST), total bilirubin (TBIL), and direct bilirubin (DBIL), were extracted by two reviewers. The Cochrane tool was used for the assessment of risk of bias included trials. Data were analyzed with RevMan 5.2.7 software. *Results*. 11 RCTs involving 1275 subjects with cholestatic hepatitis were included. Compared with essential therapy, large dosage of Chishao in formulae demonstrated more efficiently with down regulation of serum ALT, AST, TBIL, DBIL. Meanwhile, there were no obvious adverse events. *Conclusion*. As a promising novel treatment approach, widely using large dosage of Chishao in formulae may enhance the curative efficacy for cholestatic hepatitis. Considering being accepted by more and more practitioners, further rigorously designed clinical studies are required.

## 1. Introduction

Cholestatic hepatitis presents the symptoms as abnormal bile generation, secretion, and excretion that leads to the intrahepatic accumulation of bile acids (BA) and other toxic compounds with progression of liver pathology. It is one of the most common but devastating manifestations in many liver diseases, and without proper treatment, it will ultimately result in cirrhosis and hepatic failure [1, 2]. Presently, there are 350 million to 400 million HBV infected individuals worldwide, and 2 percent of them suffer from cholestatic hepatitis along the progress [3, 4]. Cholestatic hepatitis known for its severe and prolong jaundice characteristic brings heavy burden to patients and society [5]. Current therapy, aiming at symptoms and mechanism, generally takes several measures such as vitamin for adequate nutrition, albumin and plasma for supporting treatment, symptomatic treatment to cutaneous pruritus, ursodeoxycholic acid (UDCA) or adrenocortical hormone for removing jaundice, and even transplantation. However, several unsatisfactory aspects and side effects could be seen in clinics, and the alternative medicine or treatment is more necessary.

Along with development of medical science, complementary and alternative medicine displays its unique role in hepatitis treatment [6–8]. Traditional Chinese medicine (TCM), known for its holistic concept and treatment based on syndrome differentiation, emphasizes formulae employment more than single drug use in treatment of diseases. Increasing current studies demonstrate that formulae with definite efficacy for specific disease regulate function of multiple organs and tissues in a way of network [9, 10]. In TCM, Yin Chen Hao Decoction has been widely used in treatment of jaundice. However, Yin Chen Hao Decoction usually fails in severe jaundice with the symptom of blood stasis. Chishao, owing to its function of blood invigoration, has been used in severe cholestatic hepatitis for decades and obtained satisfactory effects. Chishao, the dried root of* Paeonia lactiflora *pall or* Paeonia veitchii *Lynch, has been used for blood stasis and hyperactivity of liver-Yang in more than 2000 years. In 1983, Wang et al. proposed a novel approach of severe jaundice treatment by using large dosage of Chishao [11]. Following clinical studies, it suggested significant effect of Chishao in large dosage for cholestatic hepatitis [12]. Besides, Chishao in large dosage could ameliorate cholestasis induced by ANIT and bile duct ligation in animals [13]. By now, it demonstrates a promising treatment to use large dosage of Chishao on severe cholestasis in clinic. However, the systematic review on Chishao used in large dosage has not been seen. Therefore, this meta-analysis of RCTs was conducted to assess the clinical value of large dosage of Chishao relevant formulae for the treatment of cholestatic hepatitis.

## 2. Materials and Methods

### 2.1. Inclusion Criteria

The inclusion criteria were as follows. (1) Randomized controlled trials (RCTs) of patients diagnosed with cholestatic hepatitis met the criteria of Viral Hepatitis Prevention and Treatment Programs, Guidance for Clinical Research on New Drugs of TCM [25, 26]. (2) Chishao with large dose (more than 25 g, 2 times larger than the prescribed maximum dose of Chinese Pharmacopeia 2010 version) serves as the main element in formulae alone or in combination with conventional therapy compared with placebo, conventional therapy as controls. (3) Outcome measurements included one or more indices as total efficacy rate, alanine aminotransferase (ALT), aspartate aminotransferase (AST), total bilirubin (TBIL), and direct bilirubin (DBIL).

### 2.2. Exclusion Criteria

The exclusion criteria were (1) reviews, nonclinical studies, and case observations, (2) no RCTs, and (3) controlled interventions with TCM therapies as other Chinese herbs or acupuncture.

### 2.3. Search Strategy

Comprehensive searches were both performed in English and Chinese databases including PubMed, Embase, Cochrane Library, Chinese Biomedical Database (CBM), Wanfang, VIP medicine information system (VMIS), and China National Knowledge Infrastructure (CNKI) from their inception to January 2014. Search terms included traditional Chinese medicine, Chishao,* Paeonia lactiflora *pall,* Paeonia veitchii *Lynch, large dose, hepatitis, cholestatic hepatitis, cholestasis, randomized controlled trial, and clinical controlled trial.

### 2.4. Data Extraction and Risk of Bias Assessment

Data extraction and quality assessment were independently performed by two researchers (Ma Xiao and He Xuan) and disagreements were resolved by consensus. Detailed data as study design, participants' information, interventions, outcome measures, and adverse event report were extracted to a conclusive table. Assessment of symptom improvement was based on Guidance for Clinical Research on New Drugs of TCM or Disease Diagnosis and Efficacy Standards of Chinese Internal Medicine.

Cochrane risk of bias tool was used to assess methodological quality of included RCTs. There are six domains including random sequence generation (selection bias), allocation concealment (selection bias), blinding of participants and personnel (performance bias), blinding of outcome data (attrition bias), incomplete outcome data (attrition bias), and selective reporting (reporting bias). The judgment was marked as “high risk,” “unclear risk,” or “low risk.” Trials that met all the criteria were categorized as high risk of bias, whereas those that met none of the criteria were categorized as low risk of bias. The others were classified as unclear risk of bias if insufficient information was available to make a judgment.

### 2.5. Data Analysis

Statistical analysis was performed by Cochrane RevMan 5.2.7 (Cochrane Collaboration). Dichotomous data were presented as risk ratio (RR) and continuous variables as mean difference (MD), with 95% confidence intervals (95% CI). Statistical heterogeneity was assessed by Cochrane's *Q* test. Data with low heterogeneity (*P* ≥ 0.10 and *I* ^2^ ≤ 50%) were performed as fixed-effects model whereas others performed as as random-effects model. A funnel plot was used for assessing the potential publication bias.

## 3. Results

### 3.1. Characteristics of Included Trials

**Figure 1:**
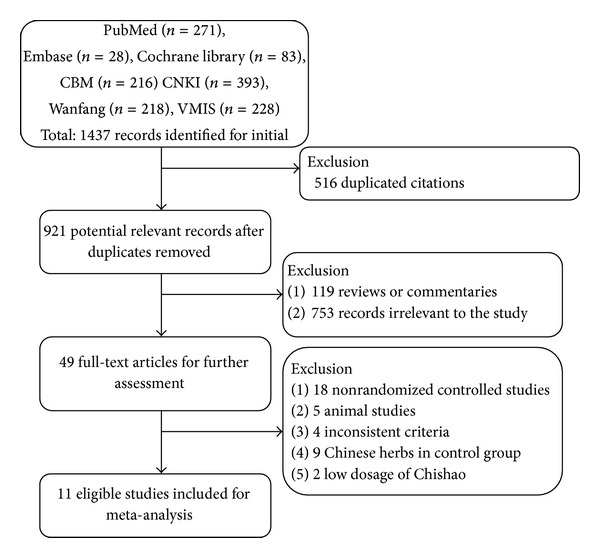
Flow diagram showing the trial selection process for the systematic review.

A total of 1437 publications were identified for initial screen and 516 duplicated citations were removed. In the remaining 921 reports, 119 reviews/commentaries and 753 records irrelevant to the study were excluded. 49 full-text articles were used for further assessment. Among them, 18 nonrandomized controlled studies, 5 animal studies, 4 inconsistent criteria, 9 Chinese herbs in control group and 2 low dosage of Chishao were excluded. The rest of 11 eligible studies were conformed to meta-analysis (Figure 1).

11 articles involving 1275 subjects with cholestatic hepatitis (751 cases in trials group, 524 cases in control group) were included in this study, and there was no significance in ages, sex, and course of disease between two groups (Table 1). Essential treatment with large dose Chishao formulae or single Chishao formulae was used in trials group, whereas essential treatment or single western medicine was applied in control group. 10 of 11 articles described treatment duration that ranged from 30 days to 2 months [14, 16–24] and 4 reported follow-up period from 2 weeks to 6 months [21–24]. Six articles reported advent events with or without slight side effect [14, 18, 21–24] (Table 2).

### 3.2. Methodological Quality of Included Trials

**Figure 2:**
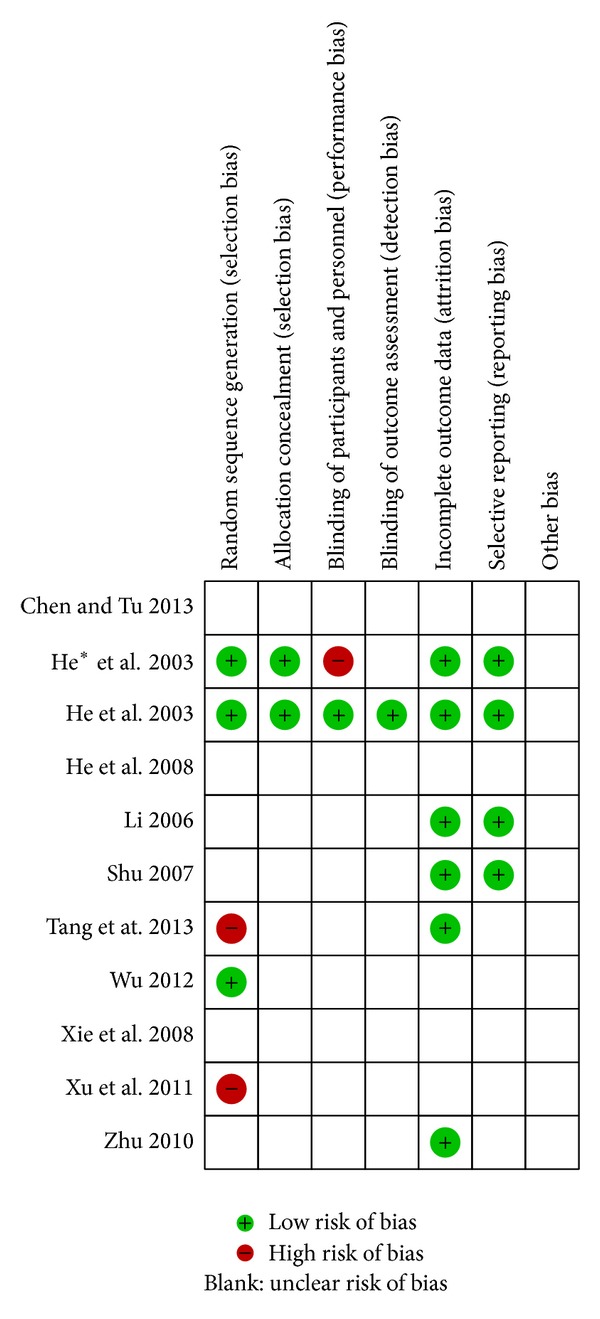
Methodological quality assessment for risk of bias for each included study.

According to Cochrane risk of bias estimation, randomized allocation of participants was mentioned in all trials; however, 3 trials reported randomized methods [16, 23, 24] through number table and 2 through clinical sequence [14, 17]. Allocation concealment was performed in 2 of 11 articles [23, 24] and 1 mentioned blinding of participants and personnel and blinding of outcome assessment [23]. There were no incomplete outcome data or essential data missing in 6 reports [14, 18, 21–24], whereas others were unclear. Four articles mentioned systematic project in research [21–24] and others remained unclear (Figure 2).

### 3.3. Outcome Measures

#### 3.3.1. Formulae with or without Essential Treatment versus Essential Treatment

**Figure 3:**
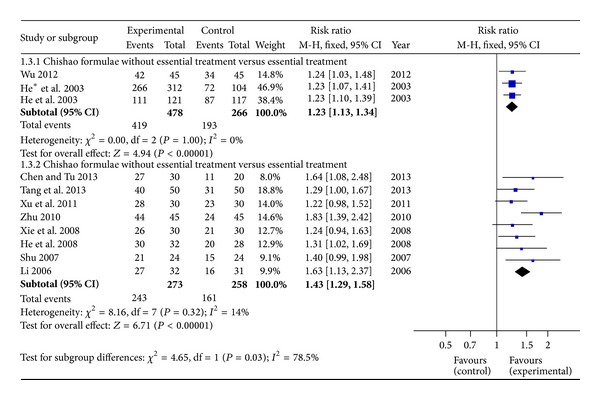
Forest plot of large dosage of Chishao relevant formulae with or without essential treatment versus essential treatment in patients with cholestatic hepatitis.

Three of 11 articles described trials group with only Chishao formulae treatment, whereas the rest 8 RCTs reported combination treatment of Chishao formulae and essential drugs in trials group. No heterogeneity was displayed in both analyses and fixed effects model was used (*P* > 0.10). The results displayed RR with 95% CI in single application and combinations were 1.23 (1.13, 1.34) and 1.43 (1.29, 1.58). It revealed that formulae with large dosage of Chishao did demonstrate an exact efficacy in cholestatic hepatitis treatment whether combined application or not (Figure 3).

#### 3.3.2. Serum Indices of Cholestatic Hepatitis Pathology

**Figure 4:**
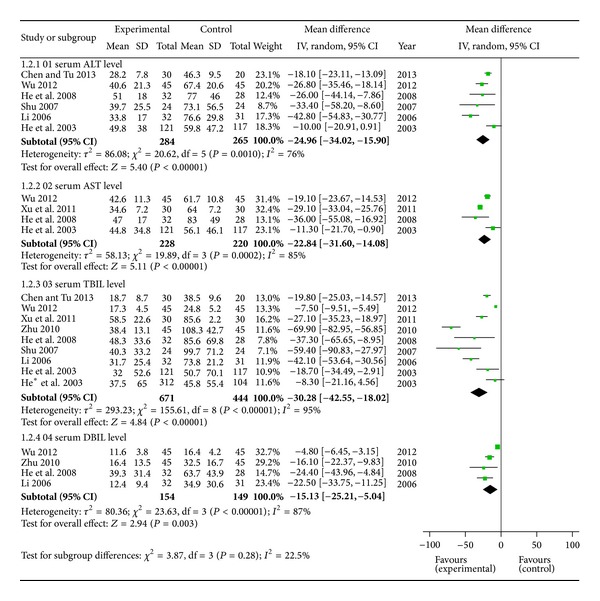
Forest plot of serum indices of cholestatic hepatitis pathology.

Serum ALT level was measured in 6 articles whereas 4 reported AST. TBIL level, the direct index of cholestatic hepatitis, was described in 9 trials and 4 displayed serum DBIL level. Heterogeneity occurred in indices of ALT, AST, TBIL, and DBIL (*P* < 0.10). Therefore, random effects model was adopted for analysis. The MDs with 95% CI of serum ALT, AST, TBIL, and DBIL level were −24.96 (−34.02, −15. 90), −22.84 (−31.60, −14.08), −30.28 (−42.55, −18.02), and −15.13 (−25.21, −5.04), respectively, indicating a significant pathologic indices decrease in trials group (Figure 4).

#### 3.3.3. Efficacy Rate of Different Course of Disease

**Figure 5:**
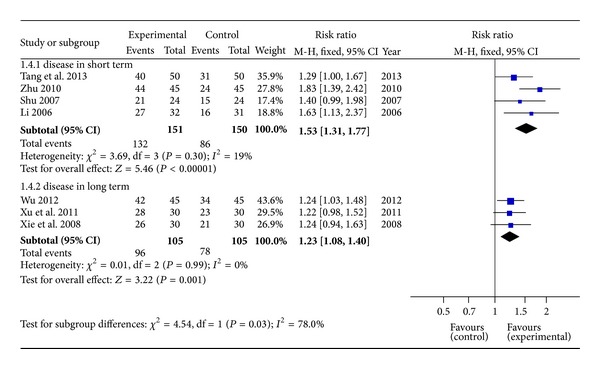
Forest plot of different course of cholestatic hepatitis.

Cholestatic hepatitis was divided into two kinds of subtype according to the course of disease. In this study, the course of cholestatic hepatitis beyond 1 year was defined as long-term disease. Otherwise, cholestatic hepatitis within 1 year was classified to short term disease. There were 4 articles ascribed as short-term subtype and 3 as long-term subtype. On the basis of fixed effects model (*P* > 0.10), both of the results showed a significant difference between trials and control. RR with 95% CI was 1.53 (1.31,1.77) in shortterm and 1.23 (1.08,1.40) in long term (Figure 5).

### 3.4. Adverse Events

Among 11 RCTs, 3 [14, 18, 21] reported no side effect occurring in clinical trials. Another 3 [22–24] observed totally 28 cases of side effect presenting as light digestive tract symptom, but there was no causality assessment for this adverse event. In spite of no difference between trials and controls, systematic safety assessment of Chishao relevant formulae needed further investigation (Table 2).

### 3.5. Publication Bias

**Figure 6:**
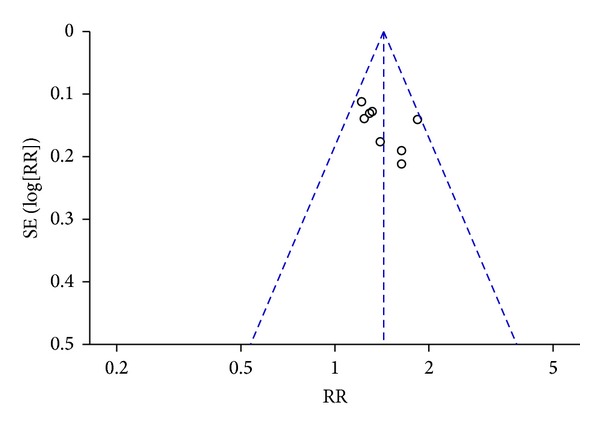
Funnel plot of large dosage of Chishao relevant formulae with essential treatment versus essential treatment in patients with cholestatic hepatitis.

A funnel plot of large dosage of Chishao relevant formulae with essential treatment verses essential treatment in patients with cholestatic hepatitis was applied with RR as the *x*-axis and SE(RR) as the *y*-axis, respectively. The plot was symmetric, suggesting that the publication bias was little (Figure 6).

## 4. Discussion

Cholestasis is defined as an impairment of bile secretion and flow followed by a lack of bile in intestine and accumulation of potentially toxic BA in the liver and the systemic circulation. Cholestasis results in intrahepatic retention of cytotoxic BA which can thus lead to liver injury or liver fibrosis [27, 28]. In cholestatic hepatitis, serum TBIL and DBIL are regarded as the sensitive diagnosis indices of cholestatic hepatitis, meanwhile, ALT and AST represent the liver function along with disease. It is well known that a variety of definite factors including infection, drug abuse, autoimmunity, heredity, gestation, and operation cause intrahepatic cholestasis. As for the mechanism of cholestatic hepatitis, more focus is localized on oxidative stress [29], inflammatory response [30], and relevant hepatocyte transporters [31, 32]. However, a systematic explanation still remains vague. At present, UDCA is recognized as the specific and potent medicine for cholestasis [33] and often combined with glucocorticoid [34]. But for long duration therapy, glucocorticoid may lead to several side effects, meanwhile UDCA will not always present stable efficacy. Transplantation, as the ultimate treatment, may result in severe immune rejection which is a devastating problem affecting quality of life. As one kind of alternative and complementary medicine, TCM shows the absolutely advantages in chronic diseases treatment. The integrated modulation to disease always involves in network style distributed to targets of molecules, tissues, and organs level [35, 36]. There are a variety of herbs and formulae in TCM that represent obvious effect of removing jaundice. Although different herbs and formulae may display different mechanisms [37], the ultimate manifestation in macro view reaches the same.

Chishao serves as an important drug of TCM. The main components are monoterpene glycosides, flavonoids, tannins, and phenolic acids [38, 39]. The herb and its active component consistently exhibit many pharmacological effects such as vasodilatation of thoracic aorta [40], antiallergic effect [41], liver protection [2], anti-inflammation, and immunoregulation [42]. However, application of large dosage of Chishao treating cholestatic hepatitis is a novel clinical practice in the recent 30 years [11]. Due to its unique efficacy, the therapeutic form is accepted by increasing practitioners. But from the safety and reasonable aspect, it brings about an essential question in TCM. Why can Chishao or even others as* Rheum palmatum* (Dahuang) and Coptis (Huanglian) be applied with such a large dosage? A recent study may reveal this answer related to the fact that different organic status represent different tolerance to drugs [43]. An experiment of* Rheum palmatum* extraction (RE) also indicates that RE, ranged certain dose, displays curative function in fibrosis animal but induces liver damage in normal [44]. Therefore, tolerance of pathologic status is relatively higher than normal one. Except that, the metabolism characteristic of Chishao is possibly another reason. The bioavailability of paeoniflorin and albiflorin in Chishao is also distinct, manifested as normal rats' bioavailability being lower compared with cholestasis hepatitis rats' [45]. Therefore, in specific pathologic status, large dosage of Chishao shall present the specific dose-effect relationship which will not be discovered in normal condition. According to both of the assumptions and examples, organic status may be the crucial and specific point to the therapy.

This systematic review included 11 randomized controlled trials and a total of 1275 participants. Large dosage of Chishao relevant formulae served as the key subject. It demonstrated a specific and potent effect for cholestasis hepatitis. As indices of hepatic pathology, the formulae led to a significant decrease in ALT, AST, TBIL, and DBIL compared with essential treatment. In addition, trials' serum level of TBIL presented the most dramatic decline, indicating a stronger removing jaundice effect than controls. Subgroup study of formulae without essential treatment manifested potent effect and formulae combined with essential treatment did also enhance the efficacy rate. The course of disease is another important factor which affects prognosis [46]. Different course of disease was investigated. The result illustrated that formulae were effective to the disease in short term or long term and acquired stronger effect than controls. Except Chishao, other herbs such as Dahuang, Yinchen, Danshen, and Gegen may also serve as important ingredients (see Supplementary Material available online at http://dx.doi.org/10.1155/2014/328152). Effects of liver protection and jaundice removing existed in these herbs. Six articles also proved that there were no obvious severe advent events occurring in RCTs. Light digestive tract symptom was observed, but there was no causality assessment for this adverse event. From the systematic analysis, large dosage of Chishao relevant formulae exhibits a promising therapy for cholestasis hepatitis.

As the requirement for medical research in translational medicine, conventional drug discovery, as style of single target to animals, faces many challenges [47]. On the contrary, TCM presents abundant experiences in clinical treatment. Fundamental research can also be sustained or indicated through this treasure. Although clinical application is mature, the mechanism of Chishao relevant formulae's effect remains far from explicit. Therefore, further focus ought to be localized on internal relationship between the formulae and their effect through the model of “clinic-experiment-clinic.”

## 5. Conclusions

Large dosage of Chishao relevant formulae may promote the curative efficacy of cholestasis hepatitis, which is a promising novel treatment approach. Considering being accepted by widespread practitioners, more rigorously designed multicenter, double-blind, randomized, and large-scale controlled trials are required.

## Supplementary Material

Affiliated Table in supplementary information showed the study ID, formulae name, dosage of Chishao and other Chinese herbal medicine in formula.

## Figures and Tables

**Table 1:** Characteristics of included studies.

Author (reference)	Published year	Cases T/C	Age (years) Range, mean		Sex Male/female	Course of disease (years) Range, mean
Tang et al. [14]	2013	50/50	T: 24–65, 41.2 C: 23–67, 42.1		T: 37/13 C: 38/12	T: 0–0.7, 0.4 C: 0–0.8, 0.4
Chen and Tu [15]	2013	30/20	NR		T: 20/10 C: 15/5	NR
Wu [16]	2012	45/45	T: 33–68, 47.9 C: 34–66, 48.4		T: 26/19 C: 23/22	T: 1–6, 3.4 C: 1–7, 3.1
Xu et al. [17]	2011	30/30	T: 18–53, 42.2 C: 16–58, 45.3		T: 18/12 C: 20/10	T: 0.1–3 C: 0.1–3.2
Zhu [18]	2010	45/45	T: 22–75, 44.6 C: 34–68, 46.1		T: 20/25 C: 22/23	T: 0.1–0.4 C: 0.1–0.3
He et al. [19]	2008	32/28	T: 16–61, 32 C: 17–59, 31		T: 19/13 C: 16/12	NR
Xie et al. [20]	2008	30/30	T: 22–61, 38.5 C: 19–65, 42.1		T: 22/8 C: 20/10	T: 0.1–6, 4.5 C: 0–10, 4.7
Shu [21]	2007	24/24	19–53	T: 36.2 C: 37.8	T: 20/4 C: 18/6	T: 0.2 C: 0.2
Li [22]	2006	32/31	T: 37.1 C: 37.3		T: 22/10 C: 17/14	T: 0.1 C: 0.1
He et al. [23]	2003	121/117	NR		NR	NR
He* et al. [24]	2003	312/104	T: 39.8 C: 41.3		T: 233/79 C: 80/24	T: 0.1^a^, 3.1^ch^ C: 0.1^a^, 3.3^ch^

T: trials group, C: control group. NR: no report. ^a^acute cholestatic hepatitis, ^ch^chronic cholestatic hepatitis.

“∗” in Table 1 and Table 2 refers to another study different from the study of the same name and year'

**Table 2:** Intervention and outcome measures of included studies.

Study ID	Intervention		Duration/followup	Adverse events	Outcome measures
	Trials group (large dose Chishao formulae)	Control group			
Tang et al. 2013 [14]	Yin Chen Xiao Dan decoction (Chishao 30 g) + Vitamin C + glucuronolactone + diammonium glycyrrhizinate Injection	Vitamin C + glucuronolactone + diammonium glycyrrhizinate injection	30 days/NR	NO	Total efficacy rate

Chen and Tu 2013 [15]	Yin Chen Hao decoction jia wei (Chishao 60 g) + sparagin + diammonium glycyrrhizinate + vitamin C	sparagin + diammonium glycyrrhizinate + vitamin C	NR	NR	Total efficacy rate, ALT, TBIL, *γ*-GT

Wu 2012 [16]	Huo Xue Qing Dan decoction (Chishao 30 g)	ademetionine injection	2 months/NR	NR	Total efficacy rate, ALT, AST, TBIL, DBIL

Xu et al. 2011 [17]	Chishao relevant formulae (Chishao 100 g) + essential treatment	Essential treatment	Hospitalization time/NR	NR	Total efficacy rate, ALB, AST, TBIL, PT

Zhu 2010 [18]	Chishao relevant formulae (Chishao 30 g–80 g) + diammonium glycyrrhizinate + vitamin K_1_ + sparagin + ademetionine or UDCA	Diammonium glycyrrhizinate + vitamin K_1_ + sparagin + ademetionine or UDCA	1-2 months/NR	NO	Total efficacy rate, ALT, TBIL, DBIL

He et al. 2008 [19]	Chishao relevant formulae (Chishao 60 g) + diammonium glycyrrhizinate Injection + alprostadil injection + UDCA + vitamin C and vitamin BCO	diammonium glycyrrhizinate injection + alprostadil injection + UDCA + vitamin C and vitamin BCO	4 weeks/NR	NR	Total efficacy rate, ALT, AST, TBIL, DBIL, TBA, CGT, ALP

Xie et al. 2008 [20]	Chishao Tui Huang decoction (60–100 g) + diammonium glycyrrhizinate	diammonium glycyrrhizinate	8 weeks/NR	NR	Total efficacy rate

Shu 2007 [21]	Wen Li Huo Xue decoction (Chishao 25 g) + silymarine + Adenosine disodiu injection	Galle Donau + silymarine + adenosine disodiu injection	2 months/1-2 months	NO	Total efficacy rate, ALT, TBIL, GGT, ALP

Li 2006 [22]	Jian Pi Li Dan decoction (Chishao 25 g) + vitamin K_1_ + vitamin BCO + glucuronolactone	Diammonium glycyrrhizinate injection + UDCA + vitamin K_1_ + vitamin BCO + Glucuronolactone	40 days/3 months	T: 2 Cases/C: 4 Cases with light digestive tract side effect.	Total efficacy rate, TBIL, ALT, TBA, ALP

He et al. 2003 [23]	Chi Dan Tui Huang granule	Galle Donau	8 weeks/2 weeks–6 months	T: 2 Cases/C: 4 Cases with light digestive tract side effect.	Total efficacy rate, ALT, AST

He* et al. 2003 [24]	Chi Dan Tui Huang granule	Galle Donau	8 weeks/2 weeks–6 months	T: 13 Cases/C: 3 Cases with light digestive tract side effect.	Total efficacy rate, TBIL

“∗” in Table 1 and Table 2 refers to another study different from the study of the same name and year'
