# Supplementary material for: Large Dosage of Chishao in Formulae for Cholestatic Hepatitis: A Systematic Review and Meta-Analysis
Source: Evid Based Complement Alternat Med. 2014 Jun 2;2014:328152. doi: 10.1155/2014/328152 (PMC4060395; doi:10.1155/2014/328152)
Supplement: Supplementary file 1 — Affiliated Table in supplementary information showed the study ID, formulae name, dosage of Chishao and other Chinese herbal medicine in formula. [file 328152.f1.pdf]

Affiliated Table: Supplementary information of all herbs for corresponding formulae

Study ID	Chishao Formulae	Dosage of Chishao	Other Chinese Herbal Medicine
Tang 2013	Yin Chen Xiao Dan decoction	30 g	Yinchen, Jinqiancao, Danshen, Zexie, Zhuling, Fuling, Dahuang, Chaihu
Chen 2013	Yin Chen Hao decoction jia wei	60 g	Yinchen, Zhizi, Dahuang
Wu 2012	Huo Xue Qing Dan decoction	30 g	Dahuang, Danshen, Yinchen, Mudanpi, Danggui, Zexie, Shengdihuang, Chenpi, Gancao
Xu 2011	Chishao relevant formulae	100 g	Huangqi, Taoren, Honghua, Danshen, Sanleng, Ezhu
Zhu 2010	Chishao relevant formulae	30-80 g	Yinchen, Danshen, Huzhang, Sanqi, Yujin, Jinqiancao, Shanzha
He 2008	Chishao relevant formulae	60 g	Huzhang, Yinchen, Tufuling, Maiya, Baishao, Tianjihuang, Jiaogulan, Baihuasheshecao, Chuipencao, Ezhu, Baizhu, Gancao
Xie 2008	Chishao Tui Huang decoction	60-100 g	Yinchen, Zhizi, Dahuang, Dangshen, Baizhu, Fuling, Danggui, Honghua, Taoren, Jineijing, Jiaomaiya, Jiaoshenqu, Jiaoshanzha
Shu 2007	Wen Li Huo Xue decoction	25 g	Yinchen, Fuling, Zexie, Danshen, Baizhu, Gegen, Guizhi, Chenqianzi, Baihuasheshecao, Pugongying, Yujin, Ganjiang
Li 2006	Jian Pi Li Dan decoction	25 g	Huangqi, Dangshen, Zhizi, Baizhu, Jinqiancao, Yinchen, Dahuang, Chaihu, Baishao, Fuling, Haijinsha, Ezhu, Nvzhenzi, Zhigancao, Gualou, Whitmania pigra Whitman, Tabanus
He 2003	Chi Dan Tui Huang granule	Original formulae is 150 g	Danshen, Gegen, Gualou
He* 2003	Chi Dan Tui Huang granule	Original formulae is 150 g	Danshen, Gegen, Gualou
